# Reliable processing of graphene using metal etchmasks

**DOI:** 10.1186/1556-276X-6-390

**Published:** 2011-05-18

**Authors:** Shishir Kumar, Nikos Peltekis, Kangho Lee, Hye-Young Kim, Georg Stefan Duesberg

**Affiliations:** 1 School of Chemistry, Trinity College Dublin, Ireland; 2 Centre for Research in Nanostructures and Nanodevices (CRANN), Trinity College, Dublin, Ireland

## Abstract

Graphene exhibits exciting properties which make it an appealing candidate for use in electronic devices. Reliable processes for device fabrication are crucial prerequisites for this. We developed a large area of CVD synthesis and transfer of graphene films. With patterning of these graphene layers using standard photoresist masks, we are able to produce arrays of gated graphene devices with four point contacts. The etching and lift off process poses problems because of delamination and contamination due to polymer residues when using standard resists. We introduce a metal etch mask which minimises these problems. The high quality of graphene is shown by Raman and XPS spectroscopy as well as electrical measurements. The process is of high value for applications, as it improves the processability of graphene using high-throughput lithography and etching techniques.

## Background

Graphene has many potential applications including micro-nanoelectronics, sensors and transparent electronics. For applications in electronics, the reliability of processing of graphene is a major obstacle. The processing of graphene requires a transfer or growth on an insulating substrate, its patterning and subsequent contacting. With recent development of large scale synthesis of graphene layers [1-4], its use in high volume applications has become a serious option. Especially suitable for electronics is large scale chemical vapour deposition (CVD) growth of graphene on metal surfaces, as good quality graphene in an acceptable thermal budget has been reported [5,6]. Recently, large-scale transfer and patterning of graphene have been shown by [7,8]. In order to fabricate graphene-based devices, lithographic patterning is used to make etch masks, using standard positive or negative resists. This is followed by oxygen-based plasma to remove graphene, and subsequent removal of the residual resist.

Each of these processing steps may affect the quality of the graphene as defects can be created, and contaminants can be introduced. While contaminants or solvent residues may be reduced by annealing and/or cleaning procedures, polymers residues are difficult to remove with these techniques. Harsh cleaning conditions may cause introduction of defects to the graphene layers or its delamination due to the absence of interfacial bonds to the substrate. Recently, the substrate effects and possible capture of contaminants under the graphene layers have been discussed [9].

In this article, we show reliable processing of graphene on insulating substrates to produce high quality graphene field effect transistor (FET) devices. CVD graphene grown on copper was used, which was analysed by various methods after transfer. The patterning of graphene is of note, as this step was found to be unreliable using conventional methods, i.e. by the use of polymers as etch masks [7,8]. In our experiments, delamination of graphene occurred when removing the mask after etch treatment. This may be attributed to the low adhesion of graphene to the substrate in absence of chemical bonds. In order to deal with this problem, we have introduced metal patterns as etchmask. The graphene is covered with a Ni mask which is later removed by non-oxidizing acids. The process flow further avoids the exposure of the active graphene layers with polymers during plasma processing, reducing the possibility of polymer residues.

## Results and discussion

**Figure 1:**
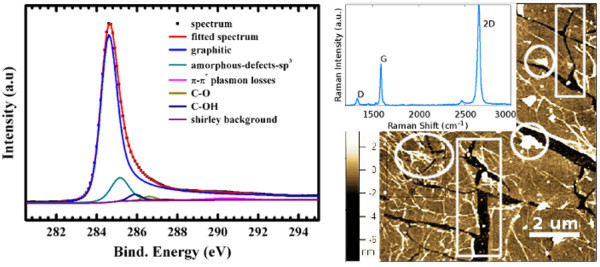
**Characterisation of as transferred graphene on SiO**_**2 **_**substrates**. On left XPS spectrum shows good quality graphene, on right AFM image shows presence of large flakes of graphene with some contamination (marked by circles) and cracks (marked by rectangles). The Raman spectrum of monolayer graphene obtained from the sample is shown in inset.

CVD-grown films on Cu of typically 1 × 1 cm^2 ^were transferred onto SiO_2 _and characterised by XPS and AFM. The XPS spectrum is shown in Figure 1 (left). The main fit has the characteristic asymmetry of graphitic structure. The second biggest fit accounts for amorphous, aliphatic and *sp*^3 ^bonds, indicating that some contaminants were residing on graphene. An AFM scan (Figure 1, right) of the graphene after transfer to the SiO_2 _shows some cracked regions and impurities on the graphene, partly in big clusters, indicating polymer fragments coming from the transfer process. The minimum height of the flakes was between 1 and 2 nm, indicating mono- or bilayer graphene. The large 2*D *(approx. 2665 cm^-1^) to *G *(approx. 1580 cm^-1^) peaks ratio shown in the inset supports this. The Raman spectrum also shows some defects as a small visible *D *peak (approx. 1350 cm^-1^). This could also indicate the presence of impurities.

**Figure 2:**
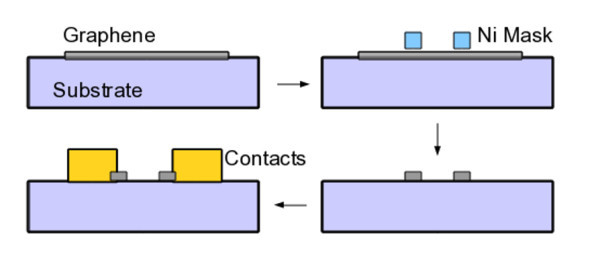
**Schematic of processing for graphene**. In first step, graphene is transferred on to suitable substrates and Ni etch mask is created on it in second step. Plasma etching and removal of Ni produced patterned graphene, which is then contacted in the last step using standard liftoff process.

**Figure 3:**
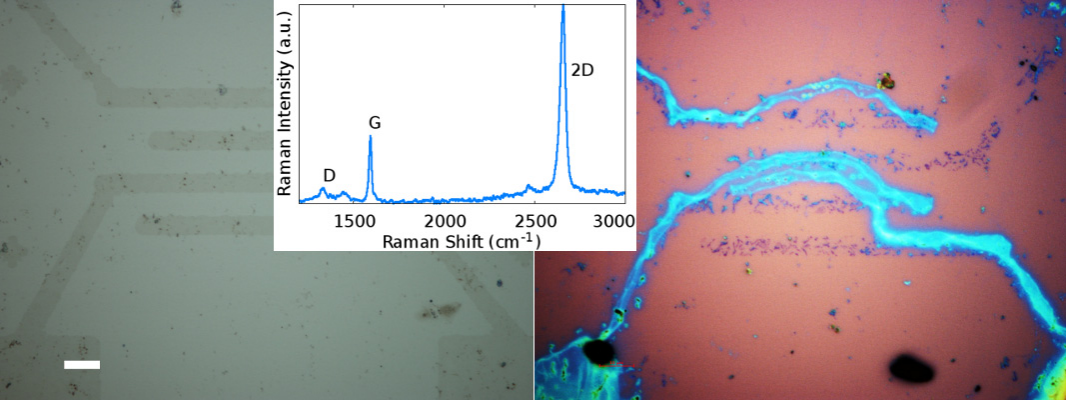
**On the right severe delamination of graphene caused by resist (blue) removal**. With metal mask no delamination occurs as shown on left (scale bar represents 10 μm). The inset shows typical Raman spectrum of etched graphene, note the smaller barely noticeable D peak (approx. 1350 cm^-1^) compared to Figure 1.

The graphene films were now patterned using optical lithography with negative resist followed by ICP plasma etch on an Oxford Instruments Plasmalab 200 in Ar/O_2 _atmosphere. The etch time ranged from 15 s to 2 min and plasma power between 100 and 500 W. The substrate holder was cooled by helium flow, and a heating of the substrate causing a possible crosslinking of the polymer resist cannot be ruled out. A 30 s treatment in a barrel asher (Diener) under O_2 _plasma was also tried. {AQ: Please supply missing word or phrase between "with" and "O_2_" in the sentence, "A barrel asher..." }A range of resist was investigated in our study which included AZ nLOF 2070 (~500 nm), maN-2403 (~ 300 nm) and S1813 (~ 1 μm). Bilayers photoresist masks using AZ nLOF 2070 as top layer and PMMA (450 k) or LOR-10B as bottom layers were also used. As delamination occurred during lift off of these resists, we created a processing procedure using a metal hard mask as shown in Figure 2. The optical images in Figure 3 depict several micron-sized structures formed using the two masks. The adhesion of photoresist to graphene after plasma treatment is stronger than the adhesion of graphene to substrate, which causes graphene to delaminate when photoresist is removed (Figure 3, right). Adhesion of Ni mask with graphene does not present this problem both because Ni removal is a chemical reaction, and interaction of graphene with Ni is not expected to be very different from its interaction with the oxide substrate. The very small *D *peak in Raman spectrum of etched graphene (Figure 3, left, inset) shows that the impurity concentration is low. This may be attributed to chemical action of acid on organic removing impurities. This observation was made repeatedly in our studies.

**Figure 4:**
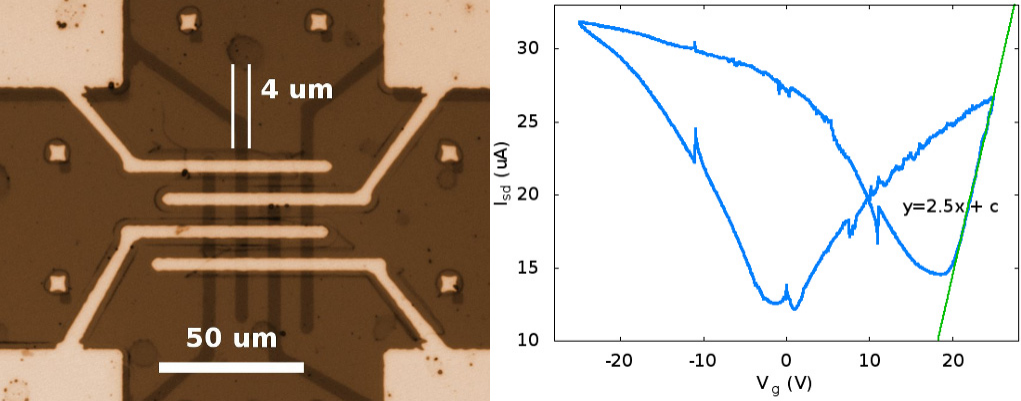
**Graphene ribbons contacted from top on Al**_**2**_**O**_**3 **_**substrates with nickel contacts**. The FET measurements were taken between two inner electrodes as source and drain, and substrate was gated from back. On the right, an *I*_sd _- *V*_g _characteristic of the device is shown at *V*_sd _of 50 mV.

The optical image of an FET device is shown in Figure 4. The width of graphene ribbons is 4 μm, which is same as the distance between the electrodes. The transistor measurements were done between the two inner electrodes, and the gate voltage was applied from the backside of the substrate. An array of devices was studied, and transistor characteristics for one of them are shown in Figure 4 (right). The charge neutrality point for the device occurs at 18 V and indicates that graphene is p-doped, presumably due to acid treatment. The slope of graph near charge neutrality points can be used to estimate the field effect mobility [10]. Using the device dimensions of *L *= 4 μm, *W *= 16 μm, transconductance, *g*_m _= 2.5 μA/V (see Figure 4), source-drain voltage, *V*_sd _= 50 mV, alumina dielectric constant ε_r _= 9.34, its thickness *h *= 60 nm, and the formula, μ_FE _= (*hg*_m_/ε_0_ε_r_)*L*/*WV*_sd_, we get μ_FE _~ 90 cm^2^/(V s). This value does not match the high values obtained from the exfoliated graphene in transistor measurements [11], but to our knowledge, the obtained mobilities are close to the highest reported using CVD graphene on oxide substrates with standard lithographic processing [12]. However, these studies have to use e-beam lithography for contacting individual flakes, which is not a scalable process. Further, in our studies, we have used substrates without optimisation of the influence of underlying trapped charges and impurities. These might be crucial, as the graphene films undergo a liquid-based transfer process. We expect that a considerable improvement can be reached when those limiting factors are optimised.

## Conclusions

CVD growth has been shown to give large area mono- or bilayer graphene. We produced FET devices arrays using optical lithography. Using metal hardmask, we were able to minimize delamination of underlying graphene films, which often occured when using standard resist. Further, the introduction of metal hardmask seems to have a minimal impact on the graphene and does prevent contamination by polymer residues. The large area processing shown here will open opportunities for graphene in industrial settings.

## Methods

Graphene was produced by CVD on copper foils at a temperature of 1000°C using methane as a carbon precursor [5]. The foils get coated with carbon on both sides. One side was spin-coated with PMMA, while on the other side, the carbon coating was mechanically removed. The foil was placed in an etchant (1 M FeCl_3_) to remove copper. The resulting film was cleaned with DI water. The graphene layer on the film was then pressed onto substrates while heated at the same time in a mechanical press. After 45 s, the support layer was dissolved in acetone and substrates were placed in chloroform for further cleaning. These substrates were then analysed with XPS, AFM and Raman spectroscopy. The XPS spectrometer used a monochromatised Al K_α _X-ray source with a resolution of 0.7 eV. For AFM an Asylum MFP-3D with standard silicon tip was employed. The Raman spectra were taken with a laser excitation of 633 nm on a Horiba Jobin-Yvon Labram spectrometer using a 100 × magnification. Silicon substrate with 300 nm of oxide on top was used for characterising transferred graphene.

Electrical measurements were done on heavily doped silicon with 60 nm of Al_2_O_3 _on top as substrates. Liftoff pattern were applied on top of graphene using a resist bilayer. Nickel (30 nm) was evaporated on the substrates and followed by a liftoff. This produced an etchmask of Ni sitting on top of graphene. The samples were then etched in O_2 _plasma in a barrel etcher to transfer the pattern on Ni to graphene underlayer. Nickel coating was finally removed using dilute HCl (1 M, 2 h). Another liftoff pattern was made on top for contacting graphene, using same process as above. Nickel contacts were deposited and a liftoff was performed again. The overall process is shown in Figure 2. The electrical characterisation of devices was done on a Suss mechanical four probe station, with Keithley 2400 sourcemeters.

## Abbreviations

CVD: chemical vapour deposition; FET: field effect transistor.

## Competing interests

The authors declare that they have no competing interests.

## Authors' contributions

SH carried out graphene synthesis and transfer, as well as SEM and Raman characterisations. NP did XPS studies. KHL and HYK conducted transport measurements and data analysis. GSD conceived of the study and participated in design and coordination. All authors read and approved the final manuscript.
